# An Exceptional Responder to Nivolumab in Metastatic Non-Small-Cell Lung Cancer: A Case Report and Literature Review of Long-Term Survivors

**DOI:** 10.1155/2019/1816472

**Published:** 2019-12-05

**Authors:** Babak Baseri, Bachar Samra, Eric Tam, Edwin Chiu, Andrea Leaf

**Affiliations:** ^1^Department of Hematology/Oncology, State University of New York Downstate Medical Center, Brooklyn, New York, USA; ^2^Department of Hematology/Oncology, Veterans Affairs New York Harbor Healthcare System, Brooklyn Campus, New York, USA

## Abstract

**Background:**

Exceptional responders to immune checkpoint inhibitors in metastatic non-small-cell lung cancer (NSCLC) are rare. Furthermore, the optimal duration of immunotherapy in patients who achieve complete remission and the benefit of rechallenge after recurrence remain unknown. Studying the clinical course of exceptional responders can help identify potential predictors of response to immunotherapy and further fine-tune our management algorithms in the absence of standard of care in challenging scenarios.

**Case Presentation:**

We highlight the case of a 73-year-old Vietnam War Veteran with active tobacco dependence who achieved complete response with nivolumab for metastatic NSCLC after four prior lines of chemotherapy. Nivolumab was discontinued after 10 cycles due to immune-mediated hepatitis that resolved with steroids. He remained in complete remission for 14 months while off therapy. Then, his tumor recurred twice locally in the mediastinum and he again achieved complete and durable responses after each recurrence with radiotherapy. Due to recurrence in both lungs one year later, he was rechallenged with nivolumab and achieved partial response after two months of therapy. He continues to do well five and a half years since his initial diagnosis of de novo metastatic NSCLC.

**Conclusion:**

Optimal management of exceptional responders to immune checkpoint inhibitors in metastatic NSCLC is largely unknown. Our case report adds to the limited data supporting the use of localized therapy for oligometastatic recurrences and rechallenge with immunotherapy for widespread disease in achieving disease control and long-term survival.

## 1. Introduction

The use of immune checkpoint inhibitors (ICI) in many malignancies including non-small-cell lung cancer (NSCLC) has revolutionized the field of oncology and has magnified the critical role of the immune system in fighting cancer [1, 2]. However, only a select group of patients derive clinically meaningful benefit from immunotherapy, ranging from improved quality of life to durable clinical responses, including rare complete remissions that may last many months even beyond immunotherapy discontinuation [3, 4]. The superior efficacy of immunotherapy in such exceptional responders has sparked an intense research interest in cancer immunobiology [1, 5]. Here, we describe the clinical course of a patient with heavily pretreated NSCLC who had an exceptional response to a short treatment course with nivolumab.

## 2. Case Presentation

A 73-year-old Vietnam War Veteran with active tobacco dependence (1.5 − 2 packs per day > 50 years), prostate cancer in remission (status post definitive radiation in 2008), and alcoholic fatty liver disease was diagnosed in November 2013 with metastatic poorly differentiated lung adenocarcinoma of the left upper lobe (LUL) with biopsy-proven pleural and pericardial metastases after he presented with pneumonia and lung nodules. Molecular studies were negative for EGFR mutation and ALK rearrangement, and nondiagnostic for ROS-1.

**Figure 1:**
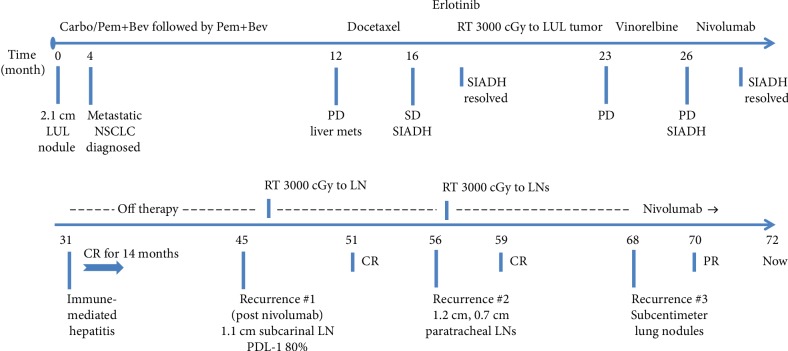
Treatment timeline. ^∗^The numbers below the axis represent the number of months since the initial presentation. Abbreviations: NSCLC—non-small-cell lung cancer; Carbo—carboplatin; Pem—pemetrexed; Bev—bevacizumab; LUL—left upper lobe of the lung; PD—progression of disease; SD—stable disease; CR—complete response; PR—partial response; LN—lymph node; PDL-1—programmed death ligand-1; SIADH—syndrome of inappropriate antidiuretic hormone secretion.

**Figure 2:**
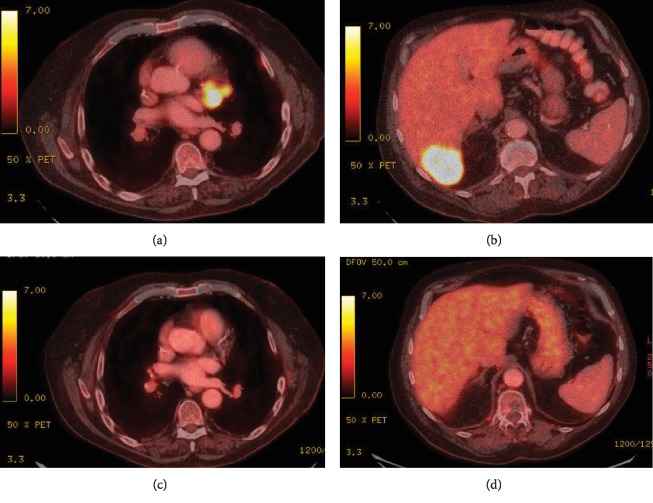
PET/CT images showing increased metabolic activity in the mediastinum (a) and in the liver (b) prior to initiating nivolumab. Resolution of FDG-avid lesions in the mediastinum (c) and in the liver (d) 10 months after discontinuation of nivolumab.

He was started on chemotherapy in January 2014 and received five cycles of carboplatin, pemetrexed, and bevacizumab, followed by three cycles of maintenance pemetrexed and bevacizumab (see Figure 1 for therapy sequence). Due to progression of disease (PD) with new liver lesions, he was switched to second-line docetaxel and he completed six cycles. Although interim positron emission tomography/computed tomography (PET/CT) showed stable disease, the patient developed a paraneoplastic syndrome of inappropriate antidiuretic hormone secretion (SIADH) during the sixth cycle, concerning for PD. Therapy was subsequently switched to erlotinib as third-line therapy. In the interim, the patient reported left shoulder pain that was attributed to a left apical lung tumor involving the pleura and was treated palliatively with RT (3000 cGy). Notably, hyponatremia resolved within one week of initiating RT, suggesting an abscopal effect given the high burden of disease outside of the radiation field. After three months of receiving erlotinib, PET/CT showed PD but the patient continued to have a good performance status. He was started on fourth-line therapy with vinorelbine and received a total of four cycles until he had recrudescence of SIADH. Imaging showed enlarging hepatic metastasis and left apical and hilar lung lesions, but no evidence of intracranial lesions. Thus, the decision was made to switch therapy to nivolumab (240 mg IV every two weeks) as fifth line. The patient received 10 cycles from August 2015 to January 2016 and his SIADH resolved after 2 months. Following four months of therapy, nivolumab was held due to grade II transaminitis, for which he was started on prednisone 100 mg daily and had a prolonged steroid taper (for six months). PET/CT following discontinuation of therapy showed no evidence of disease (NED). Nivolumab was not restarted as he was in complete remission, and it was deemed that the risks of nivolumab rechallenge outweighed its benefits. Repeat PET/CT scans (Figure 2) continued to show sustained complete remission, which lasted 14 months after discontinuation of nivolumab, until March 2017 when his tumor recurred in a 1.1 cm subcarinal node (biopsy proven, PDL-1 positive 80%; 22C3 pharmDX kit). Molecular studies showed RET rearrangement (10q11) in 84% of the cells (Leica BioSystems) and were negative for MET amplification and BRAF and HER2 mutations. Given local recurrence with very low disease burden, decision was made to treat the subcarinal node with RT (3000 cGy, 10 daily fractions of 300 cGy), with a resultant complete response. Nine months later, PET/CT showed a new hypermetabolic focus (SUV 8.7) in the right paratracheal lymph node (LN, 1.2 cm from 0.6 cm previously) and a new hypermetabolic focus (SUV 3.7) in the right middle paratracheal LN (0.7 cm). Given limited disease, RT was administered to the paratracheal LNs (3000 cGy), and complete response was again achieved until 12 months later, when his disease recurred in the lungs with new and growing bilateral subcentimeter nodules. He was rechallenged with nivolumab and has achieved a partial response after two months of therapy. He has no evidence of immune-mediated adverse events after 10 cycles. Of note, his SIADH has not recurred since the initial nivolumab therapy, and he continues to be asymptomatic with excellent performance status.

## 3. Discussion

Our case report highlights the oncologic management of a heavily pretreated patient with NSCLC who continues to be alive and well four years since his initial nivolumab treatment. The major benefit in the field of immunooncology in solid tumors, including lung cancer, has been the achievement of durable responses in a subset of patients receiving checkpoint inhibitors, with subsequent translation to longer survivals (Table 1). Most recently, the 5-year follow-up of nivolumab for pretreated advanced solid tumors (up to 5 prior lines of therapy) showed a 5-year overall survival (OS) rate of 16% across both squamous and nonsquamous NSCLC [6], compared with a historical 4% rate with traditional cytotoxic therapy [7]. Similarly, the 5-year OS rate for pembrolizumab was recently reported as 15.6% (previously treated) and 23.2% (frontline) in the Keynote 001 trial [8]. Furthermore, complete responses (CR) with ICIs are uncommon and have been reported in 1-6% of NSCLC (Table 1). A large meta-analysis of nine randomized clinical trials evaluating 4803 NSCLC patients treated with ICI reported the incidence of CR as 1.5% compared to 0.7% in chemotherapy groups [3], and found the use of ICIs as first line (relative risk (RR) 2.39, *P* = 0.032) or second line (RR 4.99, *P* = 0.038), and the use of nivolumab (RR 4.83, *P* = 0.042) and atezolizumab (RR 3.26, *P* = 0.01), but not pembrolizumab and ipilimumab, to be significantly associated with higher CR rates. However, no correlation was found between OS and CR (*r* = 0.19, *P* = 0.75). Limited data suggest that CRs may be more common in PDL-1-positive tumors and may be associated with the development of irAEs (Table 1) [9].

Predicting which patients will respond to checkpoint inhibitors is an evolving area of research. The current predictive markers of response to immunotherapy in NSCLC remain imperfect and can be divided into two main groups. The first group includes host and tumor/microenvironment factors that already exist prior to the initiation of immunotherapy. These factors include male gender [10], PDL-1 expression [11, 12], smoking status [13, 14], tumor mutation burden [15], microsatellite instability [16], presence or absence of CD8+ tumor-infiltrating lymphocytes (TILs) [11, 17], KRAS mutation or EGFR wild type [18], previous radiotherapy [19] or bevacizumab use [20], and baseline neutrophil-to-lymphocyte (NLR) ratio [21] and platelet-to-lymphocyte ratio [22]. The second group of predictors belongs to “evolving or developing” determinants that arise concurrently with immunotherapy treatment, which may be subject to exploitation by external factors. These predictive markers include but are not limited to change in gut microbiome [23], change in NLR [24] and lymphocyte-to-monocyte ratio [25], and immune-related adverse events (irAEs) [26, 27]. Based on current data, no new biomarkers have been identified in long-term survivors specifically. In the aforementioned nivolumab phase I CA209-003 trial [6], the majority of the long-term survivors were current smokers (14 out of 16 patients). In subgroup analysis, the 5-year OS rate was higher in patients with PDL‐1 expression > 50% (43% vs. 20% in PDL‐1 < 1, and 23% in PDL‐1 ≥ 1%). Most interestingly, 75% of the long-term survivors received nivolumab for no more than two years, required no further cancer therapy afterwards, and remained without disease progression as of their last follow-up visit (median follow-up of 58 months). Majority of this cohort (75%) had partial response as their best response, and none had a CR.

Case reports of exceptional responders to ICI in NSCLC are rare and possibly underreported (Table 1) [28–33]. Our case is unique in several aspects. First, it is a real-world scenario of an exceptional responder to immunotherapy outside of a clinical trial. Second, our patient has been diagnosed with metastatic lung cancer more than 5.5 years and continues to do well with very limited disease burden following eight lines of therapy, including RT. Third, he exhibited many of the predictive markers mentioned above, including high PDL-1 expression (80%), smoking history, prior RT and bevacizumab use, and development of immune-mediated hepatitis requiring nivolumab to be held. Although he continues to smoke daily, it currently remains unclear if active smoking has any role in potentiating response to ICI. Interestingly, unlike what is reported in the literature on poor responses to ICI in liver metastasis [34], our patient had a CR to nivolumab and his disease never recurred in his liver despite having a relatively large liver metastasis (Figure 2), possibly due to the activation of the immune system that resulted in hepatitis. Notably, in phase III CheckMate 017 and 057 trials, nivolumab resulted in improved OS compared with docetaxel in patients with liver metastases, and their immune-mediated hepatic adverse events were higher than the overall pooled population (10% vs. 6%) [35]. Fourth, the clinical course following nivolumab discontinuation is interesting as he achieved CR lasting 14 months after a short course of nivolumab. Notably, the prolonged course of steroid taper for his hepatitis did not affect his duration of response nor did it affect his second response to nivolumab upon rechallenge. Ongoing responses following discontinuation of immunotherapy is a well-known phenomenon and has been documented in clinical trials, including the nivolumab trial above [6, 36]. Fifth, he was noted to have a RET rearrangement on repeat biopsy. In contrast to our case, a recent case series by Offin et al. [37] showed that majority of RET-rearranged lung cancers have low PDL-1 expression and low TMB, and exhibit poor responses to ICIs. However, only one patient in that series had PDL‐1 expression > 50%, and hence no conclusions as to how to sequence targeted therapies in such patients can be drawn. In an ongoing global registry (IMMUNOTARGET), it was noted that ICI efficacy in oncogenic-driven lung cancers is inconsistent and depends on smoking history, PDL-1 expression, and the type of mutation (less benefit in RET/EGFR/ALK patients) [38]. In our case, we did not use a RET inhibitor as the patient's cancer recurrences were mostly localized, and it was thought that radiotherapy would provide the best local control with potential for greater disease-free and treatment-free intervals. Upon his last relapse, immunotherapy rechallenge was also more appealing given the patient's initial complete durable response and high PDL-1 expression. As expected, his local disease recurrences were successfully treated with radiation therapy, with responses lasting 9 and 12 months after each RT. Finally, rechallenge with nivolumab following his third tumor recurrence resulted in rapid partial response after four cycles. In the future, we plan to use a RET inhibitor as the next line of therapy once he progresses on nivolumab.

Optimal duration of immunotherapy in responders remains unclear and is an area of active investigation. A randomized trial of continuous (76 patients) vs. 1-year fixed duration (87 patients) nivolumab in advanced NSCLC (CheckMate 153, *n* = 220) showed significant improvement in progression-free survival (hazard ratio (HR) 0.42, 95% confidence interval (CI): 0.25, 0.71) and a trend towards better OS (HR 0.63, 95% CI: 0.33, 1.20) favoring the continuous nivolumab arm [39]. However, it should be noted that the continuous arm had a higher percentage of PDL‐1 expression > 50% (28% vs. 23%) and higher rates of CR (10% vs. 2%) at baseline (1-year randomization mark) compared to the 1-year fixed duration arm. Notably, two patients who achieved CR in the 1-year treatment arm recurred 6 and 13 months after stopping treatment. Retreatment with nivolumab after disease progression while off therapy resulted in response in the majority of patients, although the duration of response to the rechallenge was short for those with early progression (<6 months) [39].

Immune-related adverse events are major causes of ICI interruption or discontinuation, complicating the subsequent management of patients, particularly those who have achieved durable clinical benefit. irAEs have been generally associated with better ICI efficacy as well as increased overall survival [26, 27, 40]. Although the optimal oncologic management of patients who discontinue therapy due to irAEs is unknown, current data [6, 28], including our case report, suggest that patients who achieve a CR are more likely to have prolonged duration of response off therapy. Whether resuming immunotherapy (if prior irAE grade ≤ 2) or adding chemotherapy in the absence of measurable metastatic disease further extends duration of response or even achieves the ultimate goal of cure is currently unknown. Limited data at this time suggest that it may be reasonable to rechallenge patients with immunotherapy upon recurrence [6, 33, 41–44], or treat oligometastatic recurrences with surgery or radiation therapy, with lower threshold to initiate systemic therapy following multiple recurrences or in those with higher burden of disease [6]. Furthermore, increased use of liquid biopsies (such as circulating tumor DNA) [45, 46], tumor markers (such as CEA, CA125) [47, 48], and blood parameters (such as NLR) [46] as biomarkers of response or relapse in ICI-treated patients will further identify early responders, discern CRs from occult disease, and guide therapy in patients with early progression.

## 4. Conclusion

Exceptional responders to immune checkpoint inhibitors are uncommon in metastatic NSCLC. Additionally, the optimal duration of immunotherapy in patients who achieve complete remission remains unknown. Our case report adds to the limited data in the management of exceptional responders following discontinuation of immunotherapy. Treatment of oligometastatic recurrences with localized therapy and rechallenge with immunotherapy may increase disease control and extend overall survival. Further understanding of tumor microenvironment and tumor/immune system interactions may help identify better predictors of response and further improve patients' outcomes.

## Figures and Tables

**Table 1:** Long-term outcomes of patients treated with immune checkpoint inhibitors in metastatic NSCLC.

	Characteristics	Outcomes and exceptional responders
*Clinical trials (5 yr data)*		
Phase 1 (CA 209-003)		
Nivolumab 2nd line; Ref. [6]	129 patients Median follow-up: 58 m	Median OS 9.9 m 5-year OS 16% (43% for PDL‐1 ≥ 50%) *For 5-year survivors:* 14/16 current or former smokers, 10/16 had received prior RT, 12/16 no further tx after stopping Nivo and without PD PDL‐1 ≥ 1% in 7, ≥50% in 5, <1% in 3 pts
Phase Ib (Keynote 001)		
Pembrolizumab 1st line or later; Ref. [8]	101 tx naïve 449 previously treated Median follow-up: 60.6 m	*Treatment naïve:* Median DOR 16.8 m; CR 3% (3 patients) Median OS 22.3 m 5-year OS 23.2% (29.6% for PDL‐1 ≥ 50%) *Previously treated:* Median DOR 38.9 m; CR 1.1% (5 patients) Median OS 10.5 m (15.5 m for PDL‐1 ≥ 50%) 5-year OS 15.6% (25% for PDL‐1 ≥ 50%)

*Clinical trials (3 yr data)*		
Phase III (CheckMate 017, 057)		
Nivolumab vs. Doc 2nd line; Ref. [35]	854 patients Median follow-up: 40.3 m	Median DOR 23.8 m CR 6% for those alive at 3 years 3-year OS: 17% (vs. 8% Doc), 8% (vs. 2%) for patients with liver metastases
Phase III (Keynote 010)		
Pembrolizumab vs. Doc 2nd line or later; Ref. [49]	1033 patients Median follow-up: 42.6 m	PDL‐1 ≥ 50%: Median OS 16.9 m (vs. 8.2 m Doc) 3-year OS: 35% (vs. 13% Doc) PDL‐1 ≥ 1‐49%: Median OS 11.8 m (vs. 8.4 m Doc) 3-year OS: 23% (vs. 11% Doc)
Phase II (POPLAR)		
Atezolizumab vs. Doc 2nd line; Ref. [50]	287 patients Minimum 3-year follow-up	Median DOR: 22.3 m (vs. 7.2 m docetaxel) 3-year OS: ITT 16.6% (vs. 10% docetaxel) IHC TC3 or IC3 37.5% (vs. 14.9%) IHC TC 2/3 or IC 2/3 21.2% (vs. 9.9%)

*Case reports*		
Nivolumab 3rd line; Ref. [28]	67 male; squamous cell ca PDL‐1 TPS > 50% TMB 87-91 Mut/Mb	Nivolumab-induced pneumonitis after 3 doses; following discontinuation of the drug, the patient continued to have complete remission for 14 months
Nivolumab 2nd line; Ref. [29]	80 male; squamous cell ca RT (3 months prior to Nivo) PDL‐1 positive > 5%	Radiation-induced pneumonitis CR after 6 cycles, ongoing after 2 years of continuous nivolumab
Nivolumab 2nd line; Ref. [31]	47 male; adenocarcinoma Current smoker SBRT to LNs while on Nivo	PR after 6 cycles; CR after 13 cycles (10 weeks after RT) Nivo discontinued after cycle 17 due to pancreatitis; still in CR almost 2 years since discontinuation

Abbreviations: OS—overall survival; m—months; tx—treatment; DOR—duration of response; CR—complete response; PD—progressive disease; ITT—intention to treat; Nivo—nivolumab; doc—docetaxel; LN—lymph node; ca—carcinoma; NSCLC—non-small-cell lung cancer; RT—radiotherapy; SBRT—stereotactic body radiation therapy; IHC—immunohistochemistry; TC—tumor cells; IC—tumor-infiltrating immune cells; Mut/Mb—mutations per megabase; PDL-1—programmed death ligand-1.
